# Access, accountability, and the proliferation of psychological therapy: On the introduction of the IAPT initiative and the transformation of mental healthcare

**DOI:** 10.1177/0306312719834070

**Published:** 2019-03-25

**Authors:** Martyn Pickersgill

**Affiliations:** Centre for Biomedicine, Self and Society, Usher Institute, The University of Edinburgh, Edinburgh, UK

**Keywords:** access, accountability, biomedicalization, health economics, psychological therapy

## Abstract

Psychological therapy today plays a key role in UK public mental health. In large part, this has been through the development of the (specifically English) Improving Access to Psychological Therapies (IAPT) programme. Through IAPT, millions of citizens have encountered interventions such as cognitive behaviour therapy, largely for the treatment of depression and anxiety. This article interrogates how this national response to problems of mental ill-health – and the problematization itself – was developed, accounted for, and sustained. By imbricating economic expertise with accounts of mental ill-health and mechanisms of treatment, IAPT has revivified psychological framings of pathology and therapy. However, it has done so in ways that are more familiar within biomedical contexts (e.g. through recourse to randomized controlled trial studies). Today, the initiative is a principal player in relation to which other services are increasingly developed. Indeed, in many respects IAPT has transformed from content to context within UK public mental health (in a process of what I term ‘contextification’). By documenting these developments, this paper contributes to re-centring questions about the place and role of psychology in contemporary healthcare. Doing so helps to complicate assumptions about the dominance of linear forms of (de)biomedicalization in health-systems.

At the turn of the century, an adult in England who felt very down, lacking in hope and unable to enjoy everyday pleasures might have made an appointment with their General (Medical) Practitioner (GP). The GP would then, perhaps, have decided that their patient was suffering from depression, with a prescription for a pharmaceutical such as a selective serotonin reuptake inhibitor (SSRI) being a likely outcome. Today, there is a reasonable chance that some kind of talking therapy would be recommended. In fact, a GP appointment is not always necessary at all: The potential patient may call a local National Health Services (NHS) therapy service, and refer themselves directly. Such services are likely to be badged as part of, or to have evolved from, something called the Improving Access to Psychological Therapies initiative (IAPT).^1^ This much-lauded public health programme has had many millions of pounds invested in it, with the objective of making treatment for mental illness more accessible (Department of Health, 2012).

The beginnings of IAPT are largely associated with the campaigning of (among others) London-based clinical psychologist David Clark and health economist Lord Richard Layard. Through IAPT, the number of clinicians delivering therapy has proliferated and ideas about the salience of the psychological within the NHS have been reshaped. During the early days of IAPT, therapists targeted men and women of working-age and focused on depression and anxiety, employing psychological interventions to treat those conditions. IAPT later began to include older adults (Ghosh, 2009), and since 2011 children and adolescents have been part of its remit (Fonagy and Clark, 2015). Services have further extended into the terrain of ‘severe and enduring mental illness’ (Jolley et al., 2015).^2^ With this significant expansion, IAPT has gained substantial media coverage in the UK and subsequently abroad; for instance, one *New York Times* article described it as ‘the world’s most ambitious effort to treat depression, anxiety and other common mental illnesses’ (Carey, 2017). An editorial in the journal *Nature* (2012) asserted that IAPT ‘represents a world-beating standard thanks to the scale of its implementation and the validation of its treatments’ (p. 473). Such comments have acted to stimulate, and have been stimulated by, significant international clinical and policy attention to and mimesis of the initiative.

Here, I examine the instantiation, operations and ramifications of IAPT. I draw on interviews with key professional and policy actors working in organizations that shape the wider field of psychological care, alongside interviews and conversations with dozens of NHS clinicians (especially psychologists). My dataset also includes an array of English, UK, and international policy papers, journal articles, clinical guidelines and charity campaign materials (from 2011 onwards). These were assembled and interrogated in order to better understand the timeline of the development and reception of the IAPT initiative, and the repertories of accountability through which it has been both celebrated and critiqued.^3^

This article focuses on some of the movements in healthcare that the IAPT initiative reflected, helped to stabilize, and further propelled. I argue that the programme has helped to revivify psychological framings of pathology and therapy, and consolidated the role of economic expertise in adjudicating the nature and treatment of mental ill-health. However, it has done so in ways that are more familiar within biomedical contexts (e.g. through recourse to randomized controlled trial (RCTs) studies). Consequently, it has enjoined wider shifts in the landscape of UK mental healthcare. I suggest that IAPT has changed from being one ostensibly novel component of this terrain, to become an element around which other services are constituted. More generally, this paper aims to augment the attention of Science and Technology Studies (STS) to psychological science and practice. Such a focus invites questions about the extent to which we can accept biomedicalization as a de facto process underlying healthcare systems in high-income countries.

## Minds, money and biomedicine

As Petit (2015) has argued in this journal, psychology has ‘occupied a fairly marginal place within the history of science and STS’ (p. 154). Work does of course exist, scattered across different historiographical and STS traditions.^4^ In particular, the history of psychoanalysis has long been a thriving field (e.g. Hale, 1971; Sulloway, 1992; Turkle, 1978); further, there is also new interest in the historical dimensions of more recent therapeutics (Marks, 2017) like Applied Behaviour Analysis (ABA; Kirkham, 2017) and Cognitive Behavioural Therapy (CBT; Marks, 2012).^5^ Still, there remains a need for STS scholars to produce ‘greater specificity about psychology’s impact’ (Petit, 2015: 147; see also Stark, 2018).

This article represents one of many possible responses to Petit’s enjoinment, through the analysis of the origins (and impacts) of IAPT. The attention to IAPT that this article provides is, I think, particularly timely with regards to the possibility it offers for reflecting on key issues often considered to underpin contemporary healthcare in a range of nations. These are, first, the role of the economic matters in shaping care and treatment, and, second, the process of biomedicalization (Clarke et al., 2010).

In the case of the former, economic concerns shape healthcare innovation, design and delivery (Conrad, 2005). Economic expertise itself has also come to be increasingly advocated for and ultimately leveraged within health systems (e.g. in the UK). This has involved the reformulation of administrative challenges and questions for managers and clinicians in economic terms (Ashmore et al., 1989; Mulkay et al., 1987). Accordingly, the assessments of health economists can both expand and limit the availability and accessibility of different treatments (e.g. Moreira, 2011). Health economic analyses of ill-health and treatment options now have a kind of ‘evidentiary charisma’ (in the terms of Kelly, 2018) within political frameworks that orient policy towards enjoining various kinds of financial accountability and reify this as a public and moral good. In this paper, I show how economistic approaches to accounting for the efficacy of psychological therapy significantly shaped understandings of its public health and clinical value, and the technicalities of its application. Through such conceptions, economic knowledge became imbricated within the ontologies of the IAPT programme and the psychopathologies it sought to address.

Biomedicalization (Clarke et al., 2003, 2010) can be summarized as the processes by which health and illness come to be defined by and intervened in through explicitly biomedical praxis. Within the framework of biomedicalization, a key conceptual emphasis is the embroilment of medicine with technoscientific ideas and innovation (cf. the notion of medicalization). Influential within STS, sociology, anthropology, and beyond, Clarke et al. grounded their initial empirical work and theorization primarily within the US. Subsequent scholarship has deployed the concept through case-studies in other nations, including the UK (e.g. Carter et al., 2018; Norman et al., 2016; Young et al., 2016). Most studies of biomedicalization have examined its emergence and implications; fewer have considered the ‘countertrends and complications’ that Clarke et al. (2010: 15) argued are important to note. The rise of IAPT neither straightforwardly supports nor refutes theories of biomedicalization; instead, I suggest that it signals there is more to contemporary healthcare.

Some of the shifts this paper documents are powered through practices of problematization. By this I mean the dynamic and intertwined processes of defining and tackling ‘problems’ (Callon, 1986). In what follows, I demonstrate the particularly economic problematization undergirding IAPT. Following its success, the initiative has been (held to be) a model for other mental health services, and helped to legitimize bodies that have the capacity to shape healthcare (e.g. the National Institute for Health and Care Excellence, NICE). The instantiation of IAPT has thus informed wider policy and practice in the UK and beyond. My thinking on this draws on the work of MacKenzie and Pardo-Guerra (2014), in which they narrate ‘the story of Island – a new electronic platform for the trading of shares’ (p. 153). MacKenzie and Pardo-Guerra (2014) showed how what ‘was originally a ‘micro’ development on the fringes of US markets’ came to have considerable import such that ‘within little more than a decade key features of Island became close to compulsory’ (p. 153). As MacKenzie (2015) has elsewhere described, Island thus shifted from being ‘content to context’. Here, I call such a process ‘contextification’, and indicate how IAPT has been contextified within the UK. Today, it is a significant entity within mental healthcare, producing new standards against which other ventures can be held to account.

## Problematizing access

As noted above, IAPT can be largely traced to the actions of two individuals: David Clark and Richard Layard. Currently a professor at the University of Oxford (previously King’s College, London), Clark is prominent for his research around anxiety disorders and as a key innovator of a psychological treatment called cognitive behavioural therapy (CBT). This is aimed at changing how people think and feel about situations and individuals that, for instance, make them feel depressed or anxious. Layard was a professor at the London School of Economics and Political Science (LSE), and is a very influential economist. He remains a member of the Labour Party in the elite UK House of Lords, and has played major policy roles around mental health and wellbeing. First meeting at a British Academy event in 2003, Clark and Layard came to work together to try and move access to therapy further up the policy agenda (Evans, 2013a, 2013b; Layard and Clark, 2014). To do so, it seems that they actively sought to expand their own influence regarding how issues of access and therapy should be understood and addressed. Their research and campaigning found favour within the Department of Health and at Downing Street, helping to impel reconfigurations of the matrix of clinicians, services, guidelines, treatment sites, and more that comprise English mental healthcare. Ultimately, they played vital parts in orchestrating the development and roll-out of IAPT.

The campaigning of Layard and Clark did not, of course, emerge de novo. In the 1990s and during the early 2000s, many patients and clinicians were troubled by ever more testimonies that people experiencing mental ill-health were not receiving the care they needed (or that waiting times were shockingly long). Policymakers took this issue to be important, with admission to services noted in (for instance) a major NHS policy document, ‘A National Service Framework for Mental Health’ (Department of Health, 1999). This also stated a need to expand the numbers of professionals competent to deliver mental health interventions. Wider concerns were furthermore emerging about the economic ‘costs of mental illness’ (Sainsbury Centre for Mental Health, 2003: 1). Layard proved adept at steadily entwining these discourses of access and economy, focusing on depression specifically, and ultimately delimiting a straightforward framing of the (economic) problem(s) of access. In the early 2000s, he was an active publisher across a range of discursive spheres and his widely reviewed trade book, *Happiness: Lessons from a New Science* (Layard, 2005b), argued that depression was a social problem that could be addressed through psychological therapy. Following the publication of *Happiness*, Layard also wrote various clinically oriented articles (e.g. Layard, 2006a) and was a regulator discussant with journalists and policymakers, talking on related issues. Through such activities, Layard worked to at once characterize the nature of the problem of access, and to articulate its solution (cf. Callon, 1986: 196).

Layard’s messaging generated policy traction. For example, in January 2005, he was invited to present a report to the Prime Minister’s Strategy Unit, in a seminar at which Clark was also present (Evans, 2013b), on ‘Mental Health: Britain’s Biggest Social Problem?’ (Layard, 2005a).^6^ This document claimed that mental illness ‘imposes heavy costs on the economy’, asserting (in bold typeface): ‘There are now more mentally ill people drawing incapacity benefits than there are unemployed people on Jobseeker’s Allowance’ (Layard, 2005a: 1). An apt response, Layard argued (like the Department of Health before him), was a massive expansion of the mental health workforce.

Senior Labour figures seemed to have been receptive to Layard’s arguments: Following his presentation, The Labour Party (2005) Manifesto committed to improve mental health through ‘behavioural [i.e. psychological] as well as drug therapies’ (p. 65). The phrasing of the manifesto promise presented psychological services as a kind of addition to the (pharmaco)therapeutic status quo. It thus underscored neurobiology as a key aspect of the UK mental health landscape (see Pilgrim and Rogers, 2005; Rose, 2007). Yet, the commitment also reflected increasingly outspoken voices (such as Layard’s) in support of proliferating specifically psychological therapy. As we will see, the Labour Party pledge ultimately materialized as the IAPT initiative.

Layard’s 2005 paper subsequently evolved into ‘The Depression Report’ (Centre for Economic Performance, 2006). Released on 19 June 2006, it was also widely termed ‘the Layard Report’, indicating the importance ascribed to Layard in developing IAPT. Though most associated with him, the report advertised its authority through a list of impressive-sounding signatories from mental health research and practice, including Clark. It further demonstrated epistemic accountability through its digestible number of endnotes linking the claims it made to relevant academic literature. Immediately prior to publication, Layard promoted ‘The Depression Report’ in *The Guardian* newspaper (Layard, 2006b), with the online edition linking to the LSE website where the report could be downloaded. It was further heralded on its release date in *The Observer* (the Sunday version of *The Guardian*), which later circulated a hardcopy.^7^ Such widespread dissemination enabled ‘The Depression Report’ to act as a rhetorical and collectivizing device for articulating a particular problematization of access to therapy, and expanding discussion on depression more generally (cf. Callon, 1986).

The overall message of ‘The Depression Report’ was straightforward: Depression costs the economy, it should be treated, and treatment was affordable. Economics was a key apparatus through which its normative and clinical claims were assembled. Readers were, for instance, told that ‘the total loss of output due to depression and chronic anxiety is some £12 billion a year – one per cent of our total national income’ (Centre for Economic Performance, 2006: 5; bolded for emphasis in the original). The word ‘cost’ appeared 24 times in the main text of the 14-page report, including in a section subtitle that added the prefix ‘cost-effective’ to the word ‘therapies’. Pound signs were scattered throughout the report, and – like Layard’s 2005 paper – the number of people diagnosed with mental illness receiving state benefits appeared on the first page. Even the subtitle of the report – ‘A New Deal for Depression and Anxiety Disorders’ – gestured towards the economy, implicitly reminding readers of the culturally resonant 1930s US ‘New Deal’ economic development program. Altogether, it constructed a vision of a society full of despondent individuals whose despair impacted not only themselves, but also (the wallets of) the wider citizenry.

More than any other text of the time, ‘The Depression Report’ intertwined mental health and the economy within public discourse, relating expertise associated with the latter as an essential feature of (evaluating) attempts to understand and manage the former. Such an economistic framing attracted criticism from many mental health professionals at the clinical coalface (evident across a range of blogs, newsletters, and journals). Nevertheless, it also garnered support for the therapeutic options it outlined: i.e. psychological therapy. As noted, drug treatments for and a neurobiological understanding of depression were prominent within broader popular discourse at the time (Rose, 2007), even if these were never hegemonic (Pickersgill, 2014). ‘The Depression Report’ instead urged NHS to adopt psychological therapies for redressing the economic, social, and personal costs of depression. It stated that ‘hundreds of clinical trials’ had helped to show that ‘therapy is as effective as drugs in the short-run’, and that psychological therapy ‘has more long-lasting effects than drugs’ (Centre for Economic Performance, 2006: 6, bolded in original). This promotion was ostensibly a consequence of economic calculation rather than overt ontological partisanship over what depression ‘really’ was: overall, talking therapy was presented as the most (cost-)effective solution to a pressing social problem.

In my fieldwork, psychologists and others often mentioned Layard as significant in helping to shift the mental healthcare landscape, and IAPT itself was referred to as, for instance, the ‘Layard initiative’ (respondent 2). Nevertheless, important as he was, Layard by no means acted alone. As illustrated above, his arguments took considerable cues from policy reports and charitable briefings. Aside from David Clark, Layard also worked with colleagues at the LSE (e.g. Layard et al., 2007), and the emphasis on psychological therapy within his campaign put him in dialogue with wider advocacy that operated as ‘a, kind of, pincer movement’ (respondent 6) to direct change in policy. In this regard, a range of largely London-based actors and networks were producing documents and statements about access to talking therapies. For instance, one third-sector report, ‘We Need to Talk’ (Bird, 2006), served as a de facto companion text to ‘The Depression Report’; it emphasized the effectiveness of psychological treatment, the long waiting times for accessing it, and the economic costs of lost work.^8^ A loose association of major figures in UK mental health research, policy, and practice also formed under the banner of the ‘New Savoy Partnership’.^9^ In 2007, they released the ‘New Savoy Declaration’; this called ‘on the NHS to offer appropriate psychological therapies free at the point of delivery to all people who need them’ (New Savoy Partnership, 2007). Hence, the widely circulated ‘Depression Report’ served to publicize the goals and further the momentum of a diffuse and diverse undertaking that was already beginning to gain interest and investment within Westminster.

From 2003 to 2006, an intense campaign from elite metropolitan academics and clinicians, and well-known organizations and charities, sought to move mental health higher up the policy agenda. This also related to, and was reciprocally informed by, wider governmental strategy and commitments to work and (psychological) wellbeing (Edwards and Imrie, 2008; HM Government, 2005; Rick et al., 2010). These stakeholders – among whom Layard and Clark were significant – helped to set and propel a programme for expanding access to psychological care. In doing so, they contributed, via an especially economic problematization, to the reworking of the mental health landscape from which IAPT emerged. At an early point in the campaigning, this landscape – configured through extant policy and clinical praxis – was one within which a thing called ‘depression’ could be straightforwardly delineated and operated upon by certain techniques, with those suffering from it understood to be disadvantaged by public health infrastructures that limited access to treatment. The activities of Layard and others helped to reshape it such that both economic and psychological rationalities became vital features. Depression was reified as something that should be treated primarily through psychological therapy, and which could be accounted for in terms of its economic costs (with calculations of cost also justifying psychological rather than pharmaceutical interventions). Accordingly, depression was constructed as an object not just of concern to individuals categorized as having the disorder, but to the State and the body politic more broadly.

## Remaking infrastructures and expertise

Following their commitment to improve mental health services (The Labour Party, 2005: 65), in 2006 the re-elected Labour government granted over £2 million for trialling a new programme for enhancing access to therapy within primary care: IAPT (Department of Health, 2012). A few weeks prior to the dissemination of ‘The Depression Report’ through *The Observer*, two IAPT pilots were launched, in the city of Doncaster and in Newham, East London.^10^ These were rapidly subject to various kinds of elaboration and attention. A business case was advanced in November 2006 to expand IAPT nationally, with funding confirmed in 2007 (Rick et al., 2010). By 2008, the vision of enhancing access to therapy outlined in ‘The Depression Report’ (and elsewhere) was made manifest.

Though ostensibly a bounded initiative dedicated to improving primary mental health care in England, IAPT became – in the words of one Scottish mental health policy actor (respondent 1) – a ‘*hugely* influential’ (emphasis in original) programme. This has been partly in light of the ‘fundamental economic argument’ that ‘Richard Layard and Dave Clark put forward’, and how IAPT ‘is rolling out’ and ‘the way it’s been done’. In this section and the next, I want to sketch out the contours of IAPT, and examine how its novelty and legitimacy were accounted for in order to maximize the material and symbolic investment that generated such influence. In setting out ‘the way it’s been done’, I will demonstrate how the ‘economic argument’ justifying the initiative has become tightly imbricated with the practices of care and accountability performed through the programme.

In my interviews and documentary analysis, five key features of IAPT were regularly foregrounded: the introduction of ‘self-referral’, the proliferation of therapists, the development of ‘stepped care’, a focus on evidence-based therapies, and the measurement of outcomes. To begin with, I interrogate the first three of these significant characteristics of IAPT; these comprise the infrastructural innovations of the initiative most directly geared towards its stated aims of making therapy ‘accessible’. In the next section, I attend to the design and nature of the therapeutic provision of IAPT, and how this was rendered (ac)countable. The use of evidence-based therapies and outcome measures were central to the construction, management and maintenance of (what has been often regarded as) the success of IAPT. Hence, these features played a somewhat different, though seemingly vital, role in proliferating psychological care. As we will see, they also responded to – and had ramifications for – wider UK mental healthcare.

In IAPT, people can generally contact services directly to request an appointment. This process of ‘self-referral’ was regarded as significant by many of the policy and clinical actors I encountered during my fieldwork. Still uncommon in other mental health settings, it was deemed to avoid the ‘barriers’ to accessing therapy that GPs were (and still are) often understood to impose. Self-referral was widely perceived as a ‘radical shift’ (Brown et al., 2010: 365) that would help many more individuals who understood themselves to be depressed and in need of treatment. So important was it to the leaders of IAPT that the number of patients self-referring served as a key performance indicator for services within the initiative (Department of Health, 2011). Hence, self-referral represented a means by which IAPT could simultaneously contribute to addressing its core problematic (i.e. enhancing access) and account for the extent to which this was achieved. In problematizing referral and offering a targeted solution, the architects of IAPT were further able to present their initiative to policy and clinical communities as a logical, rational endeavour with respect to the managerial and therapeutic mores of UK healthcare. Subsequently, the apparent success of the self-referral processes of IAPT has been argued to support the need for this mechanism in other UK mental health services (e.g. Brown et al., 2010).

Alongside such efforts to make care more directly accessible, the IAPT initiative greatly increased the number of health professionals able to deliver treatment, through the positioning of very many clinicians within a number of new services. A considerable proportion of the IAPT investment was spent on training several thousand cognitive behaviour therapists and new ‘psychological wellbeing practitioners’ (PWPs) (Clark, 2011: 318). Today, they work across more than 100 IAPT services, commonly delivering highly prescriptive forms of CBT – a so-called ‘manualized’ therapy. These professionals were initially tasked with treating individuals diagnosed with ‘mild to moderate’ anxiety and/or depression for a discrete (6-10) number of therapeutic encounters. Therapy was not regarded as requiring the expertise of doctorate-holding clinical psychologists, who were also more highly paid and demanded more autonomy. Hence, the training and employment of less expensive cognitive behaviour therapists and PWPs, who worked within closely defined clinical parameters, further optimized IAPTs economic outcomes.

The clinical foci and professional demographics of the IAPT workforce are thus indicative of how the economic rationalities inherent in the conception of the initiative continued to be emphasized within programme delivery. IAPT – along with other NHS policy that enjoined so-called ‘New Ways of Working’ (e.g. Care Services Improvement Partnership/National Institute for Mental Health in England (CSIP/NIMHE), 2007) – also affected figurations of therapeutic expertise and therapy. In IAPT and elsewhere, psychologists came to be decreasingly viewed as the primary professionals who should deliver psychological therapy. Rather, other psychologically-trained clinicians were able to claim sufficient expertise, assuming therapeutic protocols were closely followed. This shift helped to shape meanings of therapy beyond that delivered through IAPT. Gradually, therapy has become progressively characterized in a range of settings as a kind of pre-packaged tool that professionals, sometimes with relatively limited credentialed psychological expertise, can be trained to use in specific, pre-determined ways.

Unsurprisingly, many working in clinical psychology have lamented this widespread recasting of expertise and therapy. One senior psychologist told me how IAPT had ‘caused a lot of disquiet’ (respondent 2) – something evident during my conversations with mental health professionals – with, for instance, the ‘deskilling’ (respondent 9) of psychological services registering as a concern. In terms of the therapeutic orientation of IAPT, a policy advisor to the initiative (respondent 7) described how he had been ‘besieged’ by criticisms about its focus on CBT. With the rising prominence of IAPT and restrictions in wider healthcare funding, clinical psychologists increasingly display their clinical and economic worth (cf. PWPs, for example) by underscoring their skill in disassembling manualized therapies and recombining them on a patient-by-patient basis. This is in relation to a specific clinical formulation of a presenting problem and the psychosocial milieu inhabited by the patient. Such collective transformations in psychological knowledge and action are not reducible to IAPT; nevertheless, they have been propelled and authorized through the initiative, given its scale and the attention its architects attracted and demanded.

Connected to shifts in therapists and therapy were new institutional logics regarding the sorting out of populations suitable for therapy. Since the inception of IAPT, the care apportioned through its services has been undertaken in a self-consciously ration(aliz)ed fashion that is ubiquitously described as ‘stepped’. For example, a ‘low intensity’ CBT intervention (such as guided self-help) can be initially provided to patients, who might then be ‘stepped up’ to a more ‘high intensity’ treatment in the absence of recovery. Levels of care relate to different kinds of intervention administered by professionals with differing (kinds and degrees of) expertise. Seen as sitting ‘at the heart of the IAPT clinical model’ (Gyani et al., 2013: 605), stepped care is constituted through a conception of what we might term ‘therapeutic wastage’. It is deployed to prevent the loss of expertise, time, and, consequently, money to the treatment of mild depression, when these could be more swiftly applied and better spent (and accounted for) to and on other individuals understood to be experiencing more severe forms of distress.

IAPT did not, in fact, introduce the notion of stepped-care (see e.g. Bower and Gilbody, 2005). However, its use has proliferated through the salience of this model in what has been a very well-resourced and highly promoted programme. Many of my interlocutors worked in services that are today structured through a kind of stepped-care approach, with implications for managing patient waiting lists. This form of care generally relates closely to values of cost-effectiveness. While these have long been part of British clinical life (e.g. Department of Health, 1999), their presence has been particularly apparent since the austerity policies enacted in the wake of the 2008 financial crisis, and almost every psychologist with whom I spoke expressed anxiety regarding service capacity vis-à-vis post-2008 budget restrictions. It was around this time that the infrastructures of IAPT were being touted as therapeutically and economically effective by key clinical proponents, such as Clark (e.g. Clark et al., 2009). Stepped-care, then, represented an answer to more than one problem. Through it, economic practices of accounting and clinical practices of accountability could (co-)produce the same ends; i.e. in principle, more people could be seen for therapy at ostensibly lower costs. Stepped-care systems also resonate with a certain notion of mental ill-health, one in which severity is not merely an abstract dimension of distress, but is more or less categorical and quantifiable (e.g. ‘mild to moderate’, and ‘severe and enduring’ depression). Hence, stepped-care represents a further example of how, through IAPT, economic understandings of care provision have become increasingly enmeshed within clinical considerations.

## Knowing what works

In my interviews and conversations with policy actors, IAPT was commonly presented as a rational approach for intervening in a concrete economic, social, and clinical problem. Increasing access to psychological therapies was ‘the sensible thing to do’, as one policy advisor put it, not least because it would generate ‘a cross-ministry saving’ (respondent 10). Relating to such economic rationalism, the stated goal of IAPT was ‘to greatly increase the availability of *NICE recommended* psychological treatment’ (Clark, 2011: 318; emphasis added) – not therapy in general. NICE (the National Institute for Health and Care Excellence) is a public body that has a ‘consistent’ (respondent 12) process for the production of NHS clinical guidelines through expert Guideline Development Groups; within these, RCT evidence and assessments of cost effectiveness are salient (Moreira, 2011).^11^ Key to how the organization presents and performs accountable action are the visibility of its processes of knowledge production, and the managerial and clinical applicability, scientific veracity, and public and political palatability of its recommendations.

By framing the operations of IAPT as mandated by NICE, its advocates have been enabled and emboldened to make claims that the initiative is clinically and epistemically accountable. Through these accountabilities, the programme can then be constructed as economically and politically durable, with ‘an authority that goes beyond the authority of any particular professional group’ (respondent 10). By following NICE, IAPT could deliver ‘treatments that we *know work*’ (respondent 4; emphasis in original). Another interviewee reflected that although ‘not everyone welcomes or *accepts*’ NICE, it ‘is the only show in town, at least at this stage, to keep the investment flowing’ (respondent 3; emphasis in original). Indeed, Layard and Clark (2014) have asserted that ‘the expansion of psychological therapy would never have happened at all without NICE’ (p. 206). CBT was prominent within the 2004 (and 2009) NICE guidelines for depression, and the architects of IAPT elected to emphasize this modality in their initiative. By ascribing dominance and import to NICE, this decision-making can be reframed as no choice at all: ‘[I]n terms of a large scale programme we are almost *obliged* to go from where the evidence sits’ (respondent 3; emphasis in original).

Nevertheless, NICE is not an uncontested organization. During my fieldwork, I regularly encountered dissatisfaction about it from psychologists and other clinicians. Such discontent related to how NICE produced knowledge and subsequently guidelines, with – as one senior figure associated with the organization noted – ‘a number of people’ regarding it as being ‘too focused on CBT, to the exclusion of other psychological therapies’ (respondent 12). Concerns have been, and continue to be, advanced within peer-reviewed and more informal literatures, with frustration about the calculative style of NICE commonly articulated. One critical journal article nicely summarizes some of the concerns in its title: ‘The NICE guidelines are misleading, unscientific, and potentially impede good psychological care and help’ (Mollon, 2009). These debates are part of a wider critique of NICE (see Abraham, 2010; Moreira, 2011; Syrett, 2003), while developing a certain character through their refraction across the mental health professions. This relates especially to the occlusion of non-RCT treatment studies by the Guideline Development Groups, longstanding debates about the role diagnostic constructs (e.g. ‘major depression’) should play within psychology, and the related use of manualized therapeutic approaches targeting categorical pathologies (e.g. CBT) (Pilgrim, 2011). Despite these critiques, logics of quantification of the kind NICE is underpinned by and contributes to reifying are increasingly threaded through UK mental health praxis.

‘Evidence-based medicine’ (EBM) has long had traction within the UK (Armstrong, 2007); likewise, health economics has shaped healthcare delivery for decades (Mulkay et al., 1987). Still, these trends towards EBM and the deployment of economic expertise have certainly intensified. Early in the twenty-first century, NICE – then a young organization – operated at some distance from the everyday concerns of clinicians at the coalface of practice (cf. Hedgecoe, 2004). The first depression guidelines were only produced in 2004; prior to this, just three guidelines in mental health were available, on schizophrenia, eating disorders, and self-harm. In 2004, key players within NICE felt the need to write an article for the Royal College of Psychiatrist’s Psychiatric Bulletin introducing their organization and its activities (Kendall et al., 2004). Suffice it to say, these days NICE needs no introduction to clinicians whose practice is marked deeply by its recommendations.

The import of NICE in mental health has thus grown alongside the expansion of IAPT itself, with both increasingly interacting with one another. For example, Clark chaired the 2013 Guideline Development Group studying social anxiety disorder, the recommendations of which also emphasised a cognitive model of this construct (and subsequent treatment regimens) Clark developed with Adrien Wells (Clark and Wells, 1995; NICE, 2013: 10). Further, the perceived successes and practices of IAPT informed the evidence examined and generated by more recent NICE Guideline Development Groups (e.g. BPS/RCPsych, 2011: 80; NICE, 2011). Fifteen years since Kendall and colleagues sought to raise awareness of NICE, its dominance today inspires voluble criticism through which professionals reassert their authority to define subjective distress and therapeutic expertise in the face of the organization doing so for them, and following the expansion and trumpeting of new kinds of practice via IAPT.

With the endorsement and encouragement of NICE, CBT has come to be widely promoted and operationalized within British mental health services. During my fieldwork I asked dozens of clinical psychologists about their therapeutic practice; they almost always replied that, at minimum, they drew upon CBT approaches. IAPT itself has regularly been taken to task for its perceived (over-)reliance on this modality. One key criticism I encountered related to the notion that CBT has a good evidence base, which was framed as explaining its promotion through NICE guidelines. Critics argued that trials for therapies that already indicated effectiveness in some populations were more likely to be sponsored for further research than untested modalities. Accordingly, further evidence would be generated, with which NICE’s Guideline Development Groups might engage. In effect, knowledge about the effectiveness of CBT was presented as partly an artefact of a positive feedback loop, accelerated by the readiness to which manualized forms of CBT could be subjected to evaluation through RCTs. Again, such critiques illustrate a shifting landscape in which (particular kinds of) ‘evidence’ has come to be central to the constitution of practice; hence, the nature of that evidence is reflexively subject to uncertainty and debate.

In interviews with policymakers, policy advisers, and key clinicians, I sometimes invited reflections on the criticisms that IAPT, CBT, and NICE had attracted. These participants largely responded by presenting the widespread instantiation of CBT, the import of NICE, and the necessity of IAPT operationalizing NICE guidelines as rational and reasoned. One asserted: ‘if you think you should be trying to invest in evidence-based things then you need to be pushing the things that have evidence’ (respondent 10). A senior member of the British Psychological Society reflected: ‘if one draws on the evidence base … what else could you do?’ (respondent 2). Concerns about CBT were reframed by a senior policy actor as an indication that those working in other therapeutic traditions needed ‘to *generate* the evidence’ (respondent 3; emphasis in original) for their interventions in ways that NICE would recognise as legitimate. A more diverse array of therapies might then be included in future recommendations, and thus within IAPT. My interlocutor argued that this perspective was ‘not *unreasonable*, particularly in these straitened circumstances’. Even as evidence around CBT is decoupled from the social, technical, and economic contexts of its generation in order to justify its deployment, the reality that evidence needs to be actively made is therefore used as a means to critique the critics. This kind of shifting register was, I found, common in defences of IAPT: criticized on epistemic, economic, or humanitarian grounds, responses could readily be assembled from one of the other dimensions of the programme that reconstructed challenges as unreasonable or ill-informed.

## (Acc)Counting (for) success

Clark and Layard, as well as other IAPT leaders, have articulated the import and novelty of the initiative through health economic forecasts, the synthesis of clinical guidelines, invocations of human need, and eventual evaluation via the monitoring of clinical and socio-economic outcomes. Healthcare evaluation represents a key means of establishing intervention efficacy and demonstrating political and economic accountability (Broer et al., 2017), and has been leveraged extensively within IAPT. For instance, during the programme rollout various projects were developed to adjudicate its success, including an evaluation led by Clark and colleagues (2008) (see also Clark et al., 2009).^12^ Information-collection, codified within the IAPT Data Standard (i.e. the list of data each IAPT service must collect), continues to enable the comparison and evaluation of individual services. From April 2012, key data have been submitted monthly from IAPT service-providers to the UK Health & Social Care Information Centre (a non-departmental public body with the aim of aim of collecting comparative data to enhance quality and efficiency in healthcare). These data include demographic details about patients, the percentage of them deemed ‘recovered’, changes to their employment status, referral dates for calculating waiting times, and scores on inventories aimed at assessing (changes to) symptom severity for various conditions (e.g. the Patient Health Questionnaire, discussed below) (Clark et al., 2009; Department of Health, 2011: 19). Submitting data is itself a complicated process which involves considerable work in terms of the collection, anonymization, and authorization of the data, and decision-making with regards to who monitors and controls this. Though this mass collection of data, the effectiveness of IAPT is made (ac)countable.

Quantification can represent a solution to enduring issues of legitimacy and accountability (Porter, 1995), and patients in mental health settings have long been subject to diverse forms of calculation (Grob, 2009; Hirshbein, 2009). Within IAPT, though, the variety and consistency of data collected on patient characteristics and outcomes is commonly argued to be unprecedented. Communicated to practitioners through the ‘IAPT Data Handbook’, the rationales for this indicate the range of intertwining clinical, economic, and political uses to which metrics are put:

1. Ensure equitable use of IAPT … by people experiencing depression and anxiety disorders from all communities within the local population, actively advancing equality …
2. Actively use data collection as part of the clinical process … to enhance patients’ and IAPT workers’ engagement in collaborative decision making and treatment plan reviews …
3. Provide objective case-load and outcomes data for supervisors to enable them to review the clinical work of IAPT workers …
4. Provide IAPT workers with information that will help identify appropriate targets for intervention in the next therapy session …
5. Monitor the extent to which IAPT workers and services are providing evidence-based treatments which are consistently applied in the manner recommended by NICE
6. Measure people’s experience and benefits from IAPT services
7. Assist commissioners and service providers in monitoring and improving the quality and cost effectiveness of their services for all communities
8. Build a robust data archive to inform evolving service improvement strategies …
9. (Department of Health, 2011: 10)

The production and harnessing of data for different but connected ends also serve to represent IAPT as a departure from more traditional means of undertaking and evaluating psychological treatment. One senior psychologist involved in the programme, for instance, told me how therapy delivered outside of IAPT could be ‘very ineffective and very poorly monitored’ (respondent 8). He claimed that this ‘shouldn’t really be allowed to carry on’, given the risk of ‘adverse effects’ from poor practice. Such morally laden talk about the potential negative effects of therapy – and hence the need to scrutinize the expertise and performance of professionals – is also articulated in formal IAPT policy (e.g. Department of Health, 2011: 13). Indeed, the very fact that measures are taken at all, notwithstanding the nature of the data itself or the uses to which it is put, can be rhetorically deployed to perform the novelty and legitimacy of IAPT.

One means through which different forms of accountability come to be enmeshed in IAPT is through the employment of a tool called the Patient Health Questionnaire (PHQ-9), designed in the US in the late 1990s to screen for and assess depression (Kroenke et al., 2001). It asks a series of questions about occurrences of depressed mood. Possible responses are: ‘not at all’, ‘several days’, ‘more than half the days’, and ‘nearly every day’. These correspond to a number, with responses summed to produce an overall severity score: 0–5 is taken to indicate mild depression, 6–10 is moderate, 11–15 is moderately severe, and 16–20 is severe. Following (self-)referral to IAPT, a patient is sent the PHQ-9 to complete. The score produced confirms a patient’s suitability for the service, provides the therapist a starting point for the first appointment, and acts as a benchmark for therapeutic progress. By rendering depression quantifiable, the PHQ-9 enables judgments of severity to be deemed reasonable and therapeutic gains to be recordable, rendering clinical action accountable (to patients and service leads). Accordingly, the PHQ-9 acts as a significant apparatus for consolidating understandings of ‘depression’ as a bounded and discrete disorder. It aligns well with CBT, which as Stark (2017) has observed, intertwines “an emphasis on numerical quantification with an ordinal schema of rationality, clarity and self-fashioning” (p. 70). By configuring patent subjectivities as calculable and surveillable, the PHQ-9 also enables the evaluation of the therapist, the service, and ultimately IAPT itself.

Layard and Clark (2014) have asserted that ‘outcome measurement is probably the most important single feature of IAPT’ and that it ‘is really the only ultimate guarantee of quality’ (p. 205). Presented as descriptive statements, such comments are normative proclamations. An interlocutor closely involved with the initiative told me that one reason why IAPT was ‘successful’ was ‘because from the start’ it was decided ‘you have to actually demonstrate that what you do is worthwhile’. This meant ‘that we just *had* to be able to have outcome data on more or less everyone who was treated’ (respondent 10; emphasis in original). Outcomes measures continue to be framed as providing epistemic and procedural credibility, which translates into political accountability. Without them, as respondent 10 told me, ‘I don’t think [IAPT] would have continued political support’. Despite clinical and normative claims about the import of outcome measures in IAPT, it is the economic and (consequently) political accountabilities relating to these that leaders of the initiative have often portrayed as particularly significant.

Given the salience of outcomes monitoring to IAPT – and their subsequent promotion by initiative leaders and other key healthcare figures and institutions – it is worth noting that this practice was not introduced by the programme. Rather, monitoring and evaluation have been advocated since at least the 1990s (e.g. Department of Health, 1999; see also Roth and Fonagy, 1996). This has often been on the grounds of (political pushes towards) clinical accountability (e.g. Margison et al., 2000; National Institute for Mental Health in England (NIMHE), 2008), generating tension and conflict within therapy communities (Power, 1999). IAPT was thus constituted within a landscape where measurement was already encouraged. Nonetheless, a 2012 NHS report lamented that when ‘outcomes data is available, the variety of indicators used do not allow for effective benchmarking and comparison’ (NHS Confederation, 2012: 82). Today, calculating outcomes is more forcefully mandated by policymakers, and is key to service commissioning and provider reimbursement (so-called ‘payment by results’) in an increasingly privatized NHS (e.g. Department of Health, 2013). My interviews with mental health professionals in non-IAPT settings indicated that routine, systematic, and standardized outcomes measurements have only relatively recently become common.^13^ I was even sometimes told (e.g. by one key civil servant associated with IAPT, as well as by various psychologists) that patients ‘like’ outcome measures. Hence, outcome measures do not just ensure that the direction of accountability travels ‘up’ to service and IAPT leaders, and ultimately policymakers and politicians, but also ‘down’ to patients themselves. Given policy emphases on patient choice (Greener, 2009), augmenting accountability to service users also enhances its political legitimacy.

The implications of IAPT for mental healthcare do not, then, lie in the introduction of outcomes monitoring as a completely novel notion. Rather, the ramifications of the English IAPT initiative can be seen in how it has contributed to establishing data collection in a range of mental health services across the UK as a necessary and ever-more routine practical task for clinicians and services in order to demonstrate clinical and economic accountability. Consequently, though generally presented by IAPT leaders as appropriately responsive to the demands of technocratic governance (Porter, 1995), claims about the collection and evaluation of patient outcomes have also contributed to the consolidation of this authority.

## Discussion

A decade after its launch, the reach of IAPT continues to expand. Its operations are held up as examples from which other NHS services might learn, and policymakers and clinicians beyond the UK have expressed interest in embedding similar models within their own health systems. Australia, for instance, has self-consciously followed the lessons of IAPT (Cromarty et al., 2016). In England and the wider UK, many researchers, clinicians, and patients have praised it for helping to make the NHS accountable to scientific evidence and human need. Layard and Clark (2014) followed up the 2006 ‘Depression Report’ with a book for a general readership, *Thrive*, in which IAPT was presented as a ‘great humanitarian project’ (p. 207) and ‘has shown that it is possible in a short time to make big improvements in the lives of hundreds of thousands of people’ (p. 201). Perhaps unsurprisingly, in light of their earlier messaging, Layard and Clark (2014) described access to therapy as simultaneously ‘morally right’ and ‘vital for our economy’ (p. ix).

Still, as Barry (2002) notes, ‘the organization of any regime is always open to the possibility of contestation’ (p. 275). And criticisms of IAPT have been plentiful. Mental health professionals – including IAPT therapists and managers themselves – commonly express disquiet about the programme: to me directly in interviews and casual conversations, and across the academic and professional literature, blogs, and social media (see also Marks, 2012). Concerns relate to the widespread use of CBT (and its elite metropolitan advocates), challenges to therapeutic quality that might result from prioritizing particular kinds of outcomes measures, and how economic rationales impinge upon – and change the nature of – clinical care. Even some of the key health economic claims made about psychological therapy have been contested (McCrone, 2013), and antidepressant prescriptions continue to rise (Health and Social Care Information Centre, 2016).

When IAPT is criticized, its advocates frame the various choices made during the development of the initiative as almost inevitable. Justifications relate to the accountabilities that Clark, Layard, and colleagues have emphasized through the initiative, and the measures and means chosen to demonstrate it. The accountabilities constitutive of IAPT were, chiefly, economic, epistemic, and clinical. They were built into the operations of the programme in ways that enabled its advocates to present it as reasonable and rational, and hence any naysayers as uninformed, misguided, or even self-interested (e.g. Fonagy and Clark, 2015). Their integration with the programme has served as a robust shield with which to deflect critique. The decisions made by IAPT leaders when responding to the broader healthcare landscape during the development phase of the initiative have come, to a significant extent, to be reified as not merely *a* way of developing public mental health, but as *the* (only) way. Elegantly advanced justifications for and defenses of IAPT align closely and resonate with a seemingly ever-more economistic framing of the public good by the UK Government, further closing down spaces within which the problematizations that might propel alternative therapeutic futures can be developed. This discourse of inevitability (Massey, 1999: 7) has helped to authorize and legitimate the decisions of the early IAPT proponents and leaders, and consequently the situation of the initiative has itself morphed. From being but one feature of a wider public health ecosystem with roots in some existing practices, IAPT has reshaped its environment and is now a principal entity to which other practitioners and services must respond (cf. MacKenzie, 2015; MacKenzie and Pardo-Guerra, 2014). It is in this sense that the initiative has moved from ‘content to context’ (MacKenzie, 2015).

Such ‘contextification’ has been highly reliant on an economic problematization of ill-health. Today, the problematization emphasizes a certain kind of psychological ontology for subjective distress, the operationalizing of this in categorical terms (e.g. depression, anxiety), the possibility of swiftly quantifying such constructs in terms of severity, the necessity of standardized and readily applicable forms of therapy, and the significance of diversely trained clinicians with varying degrees of psychological expertise. This economic problematization also foregrounds the vital role of measurement in simultaneously evidencing clinical need, therapeutic efficacy, and prudent expenditure. These features are exemplified by IAPT, and while not wholly reducible to the initiative have nevertheless become ever more characteristic of other mental healthcare settings. This is in part due to the lauding that IAPT has widely received and its casting as an exemplar programme by bodies such as NICE (the influence of which itself has grown with and through the initiative).

The psychological is commonly counterpoised with the biomedical in the more polemical tropes of some mental health professionals. We might reasonably speculate whether the rise of IAPT represents a reconstitution of psychology through biomedical ways of knowing and acting upon distress – or, in contrast, a kind of dissolution of the significance of biomedicine within mental health. However, the instantiation of IAPT is neither a straightforward case-study of biomedicalization (Clarke et al., 2003) nor an example of an initiative that has evolved as direct challenge to it. IAPT has developed its character in virtue of its constitution through economic expertise that at least purports to be agnostic about the quintessence of pathology, and styles its machinations through a kind of reflexive ontological neutrality. Depending on how IAPT catches any analytic light shed upon it, either psychological or biomedical facets can reflect back – or both can. Indeed, the existence of IAPT further complicates the possibility of drawing sharp lines between these domains. Notwithstanding the wider utility of theories of biomedicalization, then, the rise of IAPT seems to signal that there is more to contemporary healthcare than analyses that are restricted to situating developments along this conceptual axis might imply.

## Notes

1. IAPT is an English, rather than a British, initiative, given the devolution of healthcare provision to the constituent nations constituting the UK. The logics associated with the initiative are pervasive beyond England, however.

2. As part of the increasingly complex interplay between the public and private sectors within England, service providers now also include organizations such as Virgin Care (part of the multinational Virgin Group) and charities such as Mind, often partnering with the NHS.

3. For the first interview phase, eleven semi-structured interviews (and one shorter, recorded conversion) were conducted, lasting between 30 minutes and two hours. One interview focussed explicitly on Scottish, rather than English, policy; however, comparisons between the two nations formed part of the discussion. A further 40 interviews with clinical psychologists in England and Scotland, and five focus groups with psychologists and advocacy organizations generated additional data. Further context is provided by very many conversations with clinicians, service-users, and activists, some of which were facilitated through public engagement events I organized or participated in. Interviews and focus groups were conducted between 2012 and 2015, with discussions with mental health professionals, service-users, activists, and others around issues of access to psychological therapy in primary care settings beginning in 2010 (and continuing to the present day).

4. For relevant work in history and STS, see Buchanan (1997), Danziger (1990), Derksen (2001), Hayward (2014), Kusch (1999), Morawski (1988), Rose (1996), and Thomson (2006).

5. Others have examined the biomedical (re)construction and treatment of psychopathology (Healy, 2004; Horwitz and Wakefield, 2007; Kitanaka, 2011; Lee, 1999; Luhrmann, 2000; Rose, 2007; Rosenberg, 2006), though have largely refrained from emphasising explicitly psychological modalities of care.

6. Clark is not cited in the report, nor listed in the acknowledgements. Rather, Baroness Molly Meacher (Layard’s wife and Chair of the East London and City Mental Health Trust) was noted as ‘a virtual co-author of the paper’ (Layard, 2005a: 25).

7. ‘The Depression Report’ was advertised in the 3rd September 2006 edition of *The Observer*, via support from the Charlie Waller Memorial Trust.

8. ‘We Need to Talk’ was commissioned by five major mental health charities: The Mental Health Foundation, Mind, Rethink, The Sainsbury Centre for Mental Health, and Young Minds. It was published one month after ‘The Depression Report’. For coverage, see Frauenfelder (2006).

9. The New Savoy Partnership included Pam Maras (then President of the British Psychological Association), Peter Fonagy (Chief Executive of the prestigious Anna Freud Centre), and Paul Farmer (Chief Executive of the leading mental health charity Mind).

10. Doncaster focussed on depression, with Newham dealing with depression and anxiety disorders. Newham was part of the geographic area covered by the East London and City Mental Health Trust, chaired by Baroness Molly Meacher – Layard’s wife, a key influence on the Layard (2005a) ‘Mental Health’ report, and a co-signatory of ‘The Depression Report’.

11. Until 2016, NICE guidelines for mental health conditions were produced by the National Collaborating Centre for Mental Health (Kendall et al., 2004).

12. An evaluation funded by the Department of Health through its National Institute of Health Research, however, raised several caveats about the initiative, not least of which related to its very swift development and implementation (see Rick et al., 2010).

13. The ‘CORE’ framework (Evans et al., 2000, 2002) was often highlighted as a useful mechanism for outcomes measurement.
